# A Three-dimensional Finger Motion Measurement System of a Thumb and an Index Finger Without a Calibration Process

**DOI:** 10.3390/s20030756

**Published:** 2020-01-30

**Authors:** Yeongyu Park, Joonbum Bae

**Affiliations:** Department of Mechanical Engineering, Ulsan National Institute of Science and Technology (UNIST), Ulsan 44919, Korea; ygpark@unist.ac.kr

**Keywords:** Wearable system, Finger motion measurement, Thumb motion, Calibration process

## Abstract

Various wearable systems have been investigated to measure hand motion, but some challenges remain. Many systems require a calibration process to map sensor signals to actual finger joint angles by the principle of measuring the length change of the finger, or bending sensors. Also, few studies have investigated how to measure thumb motion accurately using the wearable systems. This paper proposes an exoskeleton system with linear Hall sensors to measure three-dimensional hand motion without a calibration process. The calibration process is avoided by measuring finger joint angles through an absolute rotation measurement. A new wearing method with lower parts underneath the hand joints and rubber bands is proposed to fix the structure to the hand and adapt it for various hand sizes. As the thumb has a complex biomechanical feature at carpometacarpal (CMC) joint, a new measuring method of the CMC joint is proposed to directly calculate the orientation of the metacarpal. The prototype of the thumb and index finger was manufactured, and the performance was verified experimentally by using an optical motion capture system.

## 1. Introduction

Diverse hand motion measurement systems have been actively investigated for the applications of virtual reality (VR), tele-operation, biomechanics, etc. Many methods have been proposed to measure the hand motion; the most common systems are optical marker-based motion capture systems, which use infrared signals to measure the three-dimensional position of markers [1,2,3,4,5]. However, stable and robust measurement of the hand is still challenging due to occluded markers, ghost markers and dense marker sets caused by a high degree of articulation, self-similarity, and the small scale of the hand. Therefore, wearable systems have been developed in order to overcome those problems when measuring the hand motion.

They have been developed with various mechanisms: inertia measurement units (IMU) [6], infrared-based [7,8], bending sensors [9,10], soft sensors [11], etc. Perception neuron [6] can measure whole body motion using sensing modules based on IMUs; hand motion can also be measured if the sensing modules are connected to the finger. Leap motion estimates hand gestures using infrared information [7,8]. However, infrared-based systems are significantly influenced by hand gestures. For example, it is difficult to estimate the gesture of an index finger when the index finger is covered by the middle finger.

Aside from IMU- and infrared-based systems, other systems which measure the motion of the finger joint have been developed. CyberGlove [9,10] uses bending sensors which resistance is changed according to the flexed angle. Other systems calculate the finger joint angles by measuring length change of the finger: optical linear sensors [12], potentiometers and springs [13], soft sensors [11], etc. When the finger is flexed, the wrinkles on the finger joints are smoothed out, increasing the overall length of the finger. Li *et al.* developed the system by optical linear encoders [12]; the joint angle was computed from the measurement of the linear position change of a strip aligned with the finger. Our previous work used cables and potentiometers [13], so that joint angles were computed from the length change of the finger when flexing the joint, which was assessed by measuring the motion of the cables with the potentiometers. Soft materials have been also used for hand motion measurement. Park *et al.* developed a soft sensor that embedded liquid metal as a micro channel in silicon material [11], which changed resistance when it was stretched, and that, properly mounted on the finger joint, allowed the computation of the joint angle.

Those systems have the advantage that the sensor signal is not affected by external disturbances. However, they require a cumbersome calibration process. The lower sensor value when the user wears the system is different individually due to various sizes of the hand and how tightly the system is worn. The upper value is also different because of various radiuses of the finger joint. Therefore, the calibration process which maps the sensor signal to actual finger joint angle is required. As an extreme case, Cyberglove may require up to 44 different poses or guided movements [14].

Also, many wearable systems have not been taken into account measurement of a thumb motion in their design. They measured the thumb motion by properly rotating and positioning the sensing structure of the index finger on the thumb [10,11,15]. Therefore, frequently, the sensor alignment did not match the actual position of the thumb joint in real applications, resulting in low measurement accuracy [10,16]. Moreover, the thumb has additional supination/pronation (S/P) motion at the carpometacarpal (CMC) joint if flexion/extension (F/E) and abduction/adduction (Ab/Ad) axes are considered not to match the anatomical ones, which are non-orthogonal and non-intersecting [17]. However, this has been rarely addressed.

In this paper, we propose an exoskeleton system with linear Hall sensors that overcomes the limitations of the existing systems described above. The goals of the proposed system are to (1) develop a structure that can measure hand motion without a calibration process; (2) propose a new wearing method to accommodate users of various hand sizes, while the system structures remain tightly fixed to the hand; (3) develop a new measuring structure for complex three-dimensional (3D) motions of the thumb, including S/P motion.

To satisfy these goals, (1) we introduce a structure that can measure absolute rotation angles, with a compact design that does not require any calibration process; (2) a new wearing method that uses the lower parts of the hand joints and rubber bands instead of a glove; and (3) a new measuring structure to calculate the direction of the vector parallel to the metacarpal of the thumb directly, rather than to measure the finger joint angles of the CMC joint separately. The prototype of the proposed system was manufactured for the thumb and index finger, and the performance was verified using an optical motion capture system.

The characteristics of the hand and overview of the proposed system are explained in Section 2. Detailed descriptions of each component are discussed in Section 3 and Section 4. The measurement performance verification of the thumb and index finger is presented in Section 5. Conclusions and future works are summarized in Section 6.

## 2. Description of the Proposed System

### 2.1. Characteristics of a Hand

To design a hand motion measurement system, an understanding of hand features and anatomical structures is required to determine the targeted measurement range, degrees of freedom (DOFs), and accuracy of the hand motion measurement system.

**Figure 1:**
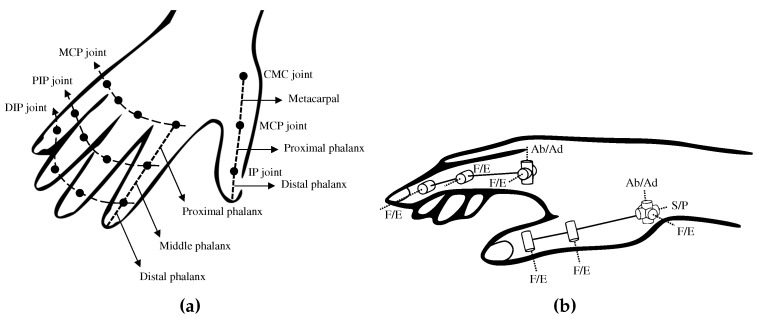
Schematics of the hand model; (**a**) Hand anatomy; (**b**) Hand biomechanical model.

The hand consists of 27 bones, 16 joints (if we consider the palm as a rigid body), and 34 muscles [18]. The finger consists of three bones: the proximal, middle, and distal phalanges; and three finger joints: the metacarpophalangeal (MCP), proximal interphalangeal (PIP), and distal interphalangeal (DIP) joints. The MCP joint has two rotational DOFs: Flexion/extension (F/E) motion in the sagittal plane and abduction/adduction (Ab/Ad) motion in the transverse plane, as shown in Figure 1. The other joints have one rotation DOF of F/E motion. The active ranges of motion (active ROM) of the MCP, PIP, and DIP joints are $-19^{\circ }∼90^{\circ }$, $-7^{\circ }∼101^{\circ }$ and $-6^{\circ }∼84^{\circ }$, respectively [19].

The thumb has the metacarpal bone, proximal, and distal phalanges. The MCP joint has two DOFs of F/E and Ab/Ad, but Ab/Ad motion is dependent on the motion of carpometacarpal (CMC) joint [20]. The metacarpal allows for three-dimensional rotation at the CMC joint beside the wrist. The CMC joint is considered to be best modeled as a two DOFs joint, with F/E and Ab/Ad axes that are non-orthogonal and non-intersecting [17,21,22]. However, if orthogonal intersecting axes are considered, a third longitudinal axis for S/P motion should be considered [23,24]. In this paper, we set the biomechanical model of the thumb as shown in Figure 1b. The CMC joint has three orthogonal axes of F/E, Ab/Ad, and S/P, and the directions of F/E and Ab/Ad axes are the same as those axes of MCP joint of the index finger. In Figure 1b, the CMC joint is slightly rotated in the direction of F/E and Ab/Ad axes.

### 2.2. Overview

**Figure 2:**
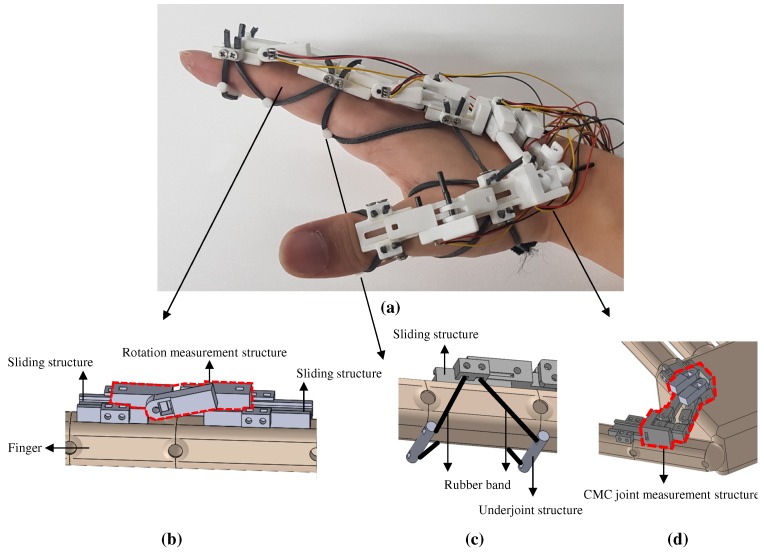
System design of the proposed device; (**a**) Prototype; (**b**) Rotation measurement structure; (**c**) Underjoint structure and wearing method; (**d**) Structure design of carpometacarpal (CMC) joint.

The prototype of the proposed system is shown in Figure 2a. Figure 2b–d show the major components of the system: a rotation measurement structure, a sliding structure, wearing configuration by an underjoint structure and rubber bands, and a CMC joint measurement structure.

The design of the rotation measurement structure is shown in Figure 2b. This structure was designed to measure the absolute angle of rotation instead of the change in the length of the finger, to exclude the calibration process. A linear Hall effect sensor (hereafter referred to as the linear Hall sensor) was used to design a compact rotation measurement structure. The sliding structure was required for the rotation measurement structure to slide over the finger phalanx as shown in Figure 2b. It had a hooked structure and the rotation measurement structure had a groove to combine with the sliding structure and moved on the sliding structure according to the rotation of the finger joint.

As shown in Figure 2c, a method was required to hold the sliding structure in place on the finger phalanx even when the finger was in motion, and to apply this for users of various hand sizes. The proposed system could fix the structure and fit various hand sizes via an underjoint structure, located under the lower part of the finger joint, and rubber bands.

For measuring the thumb motion, we propose a new mechanism to measure CMC joint motion as shown in Figure 2d. Measurement of the motion of the CMC joint is complicated. First, the CMC joint has various rotations: F/E, Ab/Ad, and S/P motions. Second, the CMC joint is attached at the wrist, such that it is difficult to place the rotation measurement structure on the CMC joint itself. In this paper, the orientation of a vector that is parallel to the metacarpal of the thumb is measured directly with respect to a frame on the dorsum rather than measuring F/E and Ab/Ad motions separately. Also, the proposed structure can measure S/P motion.

## 3. Hand Motion Measurement

### 3.1. An Index Finger

**Figure 3:**
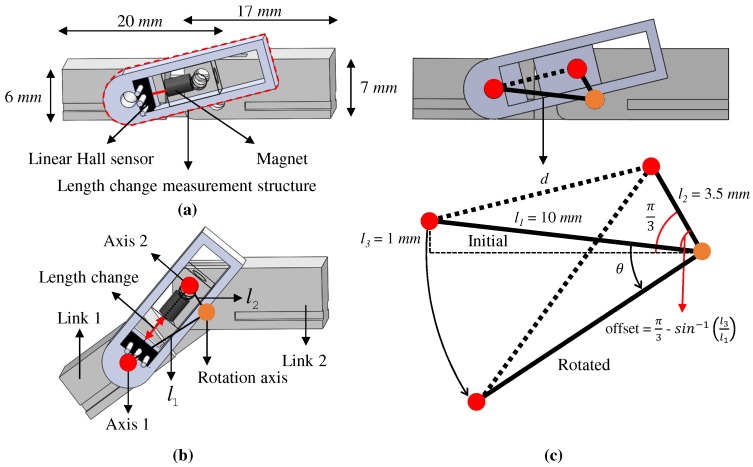
Design of rotation measurement structure; (**a**) Initial position; (**b**) Rotated position; (**c**) Calculation of rotation angle.

The design of the rotation measurement structure is shown in Figure 3. Although there are commercial sensors that can measure the absolute rotation angle, a structure using a linear Hall sensor and a magnet offers a more compact design than conventional systems, which typically include an optical encoder and a rotary Hall sensor. The optical encoder requires a disk in which the passage of light depends on the rotation angle; the minimum width of the disk is 10 mm. The rotary Hall sensor measures the absolute rotation angle by distinguishing the rotation of the sine-wave magnetic field made by the magnet which has S and N poles on the left and right sides. The rotary Hall sensor and the magnet itself are extremely small. However, a electronic board is required to house the sensor circuit, which includes capacitors and resistors. Therefore, the height of the absolute rotation angle measurement structure of conventional sensors is more than 10 mm. In the proposed design configuration, the rotation measurement structure was about 6–7 mm in height, as shown in Figure 3a, which is more compact than other commercially available systems. This is because the sensor and housing structure did not need to be located on the rotation axis in the proposed system. As shown in Figure 3a, the linear Hall sensor and magnet were installed at positions other than on the rotation axis.

The rotation measurement structure included two links and length change measurement structures consisting of a linear Hall sensor and a magnet. Links 1 and 2 were parallel to each finger phalanx adjacent to the joint to be measured, as shown in Figure 3b. The Hall sensor was attached to the structure containing Axis 1, as shown in Figure 3a, and the magnet was attached to the structure containing Axis 2. The structures (i.e., length change measurement structures) were designed so that the Hall sensor and the magnet always faced each other. The two links were connected by one rotation joint, and each link had a different rotation joint (Axis 1,2). The three rotation joints formed a triangle, and the shape of the triangle changed with the angle between the two links. The lengths from the rotation joint to Axis 1,2 were constant as $l_{1}$ and $l_{2}$, respectively, and the length connecting Axis 1 and 2 changed.

Figure 3c shows the triangular shape when the angle of rotation is $0^{\circ }$ and rotated. The formula to calculate the absolute rotation angle from the triangular shape is:

(1) $$\theta ={cos}^{-1}\left(\frac{l_{1}^{2}+l_{2}^{2}-d^{2}}{2l_{1}l_{2}}\right)-\left(\frac{\pi }{3}-sin^{-1}\left(\frac{l_{3}}{l_{1}}\right)\right)$$

where θ is the rotation angle, $l_{1},l_{2}$ are the constant lengths between the rotation axis to Axis 1, 2, respectively, and *d* is the distance between Axis 1 and 2, which is measured by the linear Hall sensor and the magnet.

The change in length was measured using the magnet and the linear Hall sensor; the distance between them was calculated by measuring the strength of the magnetic field. The distance relationship between the linear Hall sensor and the magnet was obtained experimentally, using a potentiometer. Generally, the strength of the magnetic field is inversely proportional to the square of the distance. The fitting result depicting this relationship is close to the measured value with a root mean square error (RMSE) of 0.02 V. The distance between the sensor and the magnet is as follows:

(2) $$V=\frac{\beta }{{\left(d+\gamma \right)}^{2}}+\alpha \to d=\sqrt{\frac{\beta }{V-\alpha }}-\gamma$$

where *V* is the sensor signal and *d* is the distance. α,β and γ are the fitting parameters of the mentioned relation, which were 2.43, 10.34, −1.07, respectively.

**Figure 4:**
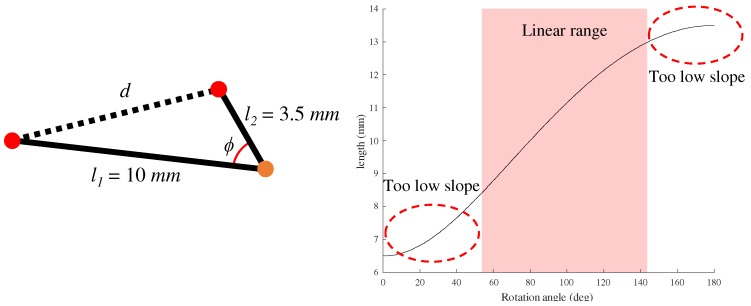
Distance (*d*) change according to the angle (ϕ) in a triangle.

In addition, the proposed rotation measurement structure has an offset of $\left(\frac{\pi }{3}-sin^{-1}\left(\frac{l_{3}}{l_{1}}\right)\right)$ when the actual rotation angle is $0^{\circ }$. The reason can be discussed in terms of the results shown in Figure 4, which shows a graphical representation of the change in the length of *d* when the angle (ϕ) between links $l_{1}$ and $l_{2}$ changes from $0^{\circ }$ to $180^{\circ }$. In the ranges $0^{\circ }∼30^{\circ }$ and $150^{\circ }∼180^{\circ }$, the change in length of *d* is significantly smaller than the change in the rotation angle. This means that a small change in length of *d* over the specified ranges can derive a large change in the angle, such that the measurement noise of the sensor is highly amplified. In the proposed design, an offset of $\left(\frac{\pi }{3}-sin^{-1}\left(\frac{l_{3}}{l_{1}}\right)\right)$ is applied to reduce amplification of sensor noise by locating the sensor signal in the linear range during the hand motion.

**Figure 5:**
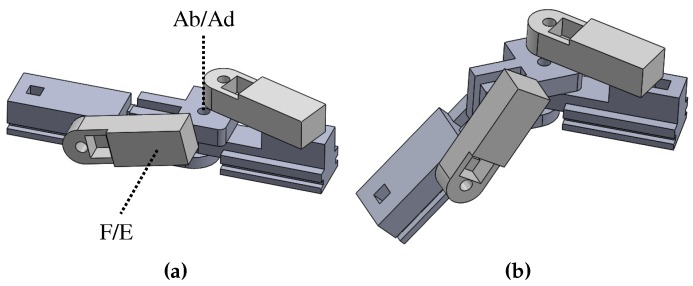
Structure design for metacarpophalangeal (MCP) joint (measurement of F/E and Ab/Ad motions); (**a**) Initial position; (**b**) Rotated.

To measure the motion of the MCP joint of two rotational DOFs, the proposed system incorporates an additional structure to measure Ab/Ad motion. As shown in Figure 5a, the additional rotation measurement structure is located perpendicular to the structure used for F/E motion. Figure 5b shows that it is possible for the proposed design to move via F/E or Ab/Ad motions, without colliding. Since the maximum angle of Ab/Ad motion is known to be $45^{\circ }$ in the left and right directions [25], the system for Ab/Ad motion is designed to allow Ab/Ad motion to move up to $45^{\circ }$ on each side.

Lastly, magnetic disturbances such as smartphones and motors might affect the sensor signal of the linear Hall sensor. However, the linear Hall sensor are not affected by those disturbances because it can only measure large magnetic field. The measurement range of the linear Hall sensor (WSH135-XPAN2) used in this system can measure around ±1000G (Gauss).

### 3.2. A Thumb

The developed rotation measurement structure was also applied to measure F/E motion of the MCP joint of the thumb; although the MCP joint of the thumb has two DOFs rotations, we only applied one rotation measurement structure of F/E motion because the Ab/Ad motion of MCP joint is dependent on the motion of CMC joint.

For measurement of the motion of the CMC joint, the proposed rotation measurement structure cannot be applied for the following reasons. First, the thumb has additional S/P motion, which makes it difficult to measure using the proposed rotation measurement structure. Second, it is difficult to fix the rotation measurement structure to the CMC joint without disturbing wrist movement, which has the rotational motion of two DOFs.

In this paper, we propose a new method to measure the CMC joint. The proposed wearing method that will be explained in next section can fix the sliding structure on the metacarpal without any restrictions on the CMC joint. Then, the vector direction, which is a combination of the F/E and Ab/Ad motions by the CMC joint of the thumb, is measured directly to calculate the positions of two points on the sliding structure.

**Figure 6:**
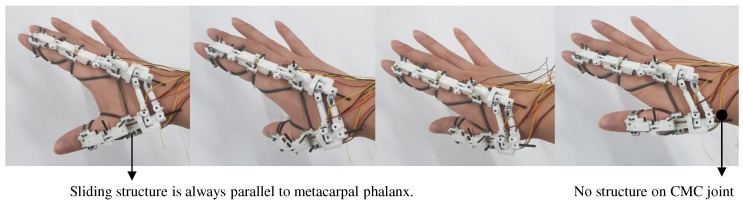
Proper structure alignment along various CMC joint rotations.

Figure 6 shows the sliding structure placed on the metacarpal of the thumb using the proposed wearing method and its movement with 3D thumb motion. Here, the user can fix the sliding structure on the metacarpal under various movements of the thumb without any restrictions on the CMC joint. Also, the vector direction of the metacarpal can be obtained from the position of the sliding structure, as the sliding structure is always kept parallel to the metacarpal.

**Figure 7:**
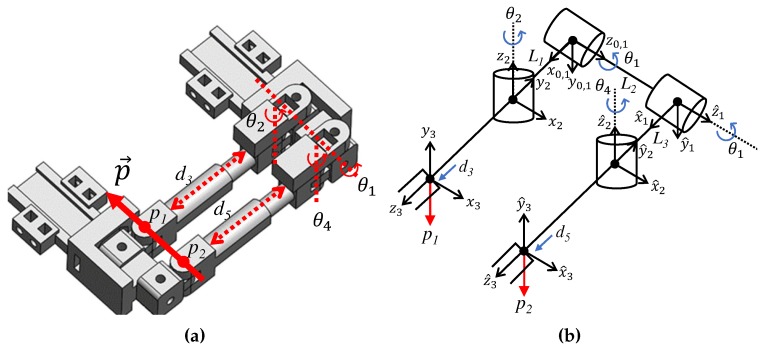
DH parameter of CMC joint structure; (**a**) System joints; (**b**) Coordinates.

The structure design to calculate the vector direction of the metacarpal and the coordinates of the Denavit–Hartenberg (DH) parameter are shown in Figure 7, and Table 1 and Table 2. The proposed system measures the absolute positions of two points of the sliding structure ($P_{1},P_{2}$) with respect to the dorsum, and obtains the direction of the vector connected by the two points. Forward kinematics by the DH parameter is used to calculate the positions of $P_{1}$ and $P_{2}$. The positions of $P_{1}$ and $P_{2}$ using forward kinematics are as follows:

(3) $$\begin{array}{ccc} P_{1,x} & = & L_{1}cos\theta_{1}+d_{3}cos\theta_{1}sin\left(\theta_{2}+\frac{\pi }{2}\right) \end{array}$$

(4) $$\begin{array}{ccc} P_{1,y} & = & L_{1}sin\theta_{1}+d_{3}sin\theta_{1}sin\left(\theta_{2}+\frac{\pi }{2}\right) \end{array}$$

(5) $$\begin{array}{ccc} P_{1,z} & = & -d_{3}cos\left(\theta_{2}+\frac{\pi }{2}\right) \end{array}$$

(6) $$\begin{array}{ccc} P_{2,x} & = & L_{3}cos\theta_{1}+d_{5}cos\theta_{1}sin\left(\theta_{4}+\frac{\pi }{2}\right) \end{array}$$

(7) $$\begin{array}{ccc} P_{2,y} & = & L_{3}sin\theta_{1}+d_{5}sin\theta_{1}sin\left(\theta_{4}+\frac{\pi }{2}\right) \end{array}$$

(8) $$\begin{array}{ccc} P_{2,z} & = & L_{2}-d_{5}cos\left(\theta_{4}+\frac{\pi }{2}\right) \end{array}$$

where $P_{i,x},P_{i,y},P_{i,z}$ represent the $i^{th}$ position in the direction of *x, y* and *z*-axes, respectively. Other parameters can be found in Figure 7.

The vector direction of the metacarpal of the thumb is given by

(9) $$\vec{P}=\frac{\vec{P_{1}}-\vec{P_{2}}}{\|\vec{P_{1}}-\vec{P_{2}}\|}$$

where $\vec{P}$ is the vector direction parallel to the metacarpal, and $\vec{P_{1}}$, $\vec{P_{2}}$ are the position vectors from the reference point on the dorsum to the positions of $P_{1}$, $P_{2}$, respectively.

Once the vector direction is determined, the F/E and Ab/Ad angles can be calculated according to a predetermined thumb joint model. In this paper, it is assumed that the F/E and Ab/Ad axes are orthogonal and are matched to the *x*- and *y*- axes of the 0*^th^* frame in Figure 7b. The F/E and Ab/Ad angles are calculated by rotating the unit vector of (0,0,−1), using Euler angles in the direction of the *x*- and *y*- axes with respect to the 0*^th^* frame, to become the $\vec{P}$ vector of Equation 9. In terms of the coordination of the CMC joint in Figure 7b, we just use simple rotation axes for validation [11] because our proposed method does not consider the anatomical rotation axes to measure the motion of the CMC joint; the orientation of the CMC joint is directly measured as a vector form.

**Figure 8:**
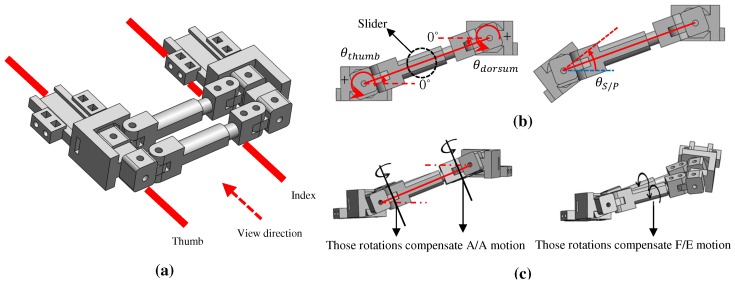
Calculation of S/P angle; (**a**) Initial position; (**b**) S/P motion when F/E and Ab/Ad motions do not occur; (**c**) Structure movement when F/E and Ab/Ad occur.

The proposed system also can measure S/P motion; the details are illustrated in Figure 8. Figure 8a shows the initial position of the CMC joint structure, and Figure 8b presents the situation when the relative positions change. As shown in the left side of Figure 8c, there are rotary Hall sensors attached to the ends of the slider that connect the dorsum of the hand and the metacarpal of the thumb to measure rotation of the dorsum and the metacarpal; these rotations are referred to as $\theta_{thumb}$ and $\theta_{dorsum}$, respectively. The reference lines of $\theta_{thumb}$ and $\theta_{dorsum}$ are indicated by dotted red lines and are set to be positive in the counterclockwise direction.

The left side of Figure 8b is the situation when the thumb structure is simply lowered, but the S/P angle is $0^{\circ }$. In this case, the reference lines of the Hall sensors on the thumb and the dorsum are parallel; thus, $\theta_{thumb}$ and $\theta_{dorsum}$ are the same as the alternate angle:

(10) $$\theta_{thumb}=\theta_{dorsum}$$

The right side of Figure 8b is the situation after S/P motion, and the rotated S/P angle is $\theta_{S/P}$. Although the S/P motion occurs in a counterclockwise direction (positive value), the measured sensor signal is negative because the sensor itself is rotated (i.e., $\theta_{thumb}=-\theta_{S/P}+\theta_{dorsum}$). From the above observations, $\theta_{S/P}$ is given by the following:

(11) $$\theta_{S/P}=\theta_{dorsum}-\theta_{thumb}$$

Notably, the equation for $\theta_{S/P}$ does not change, even when F/E and Ab/Ad motions of the CMC joint occur. The left side of Figure 8c shows the position with Ab/Ad motion. The S/P angle is not affected, because the rotation structures next to the structures of $\theta_{thumb}$ and $\theta_{dorsum}$ are rotated. In the case of F/E motion (right side of Figure 8c), the S/P angle is also not affected as the rotation axis of the slider is rotated. Therefore, the F/E and Ab/Ad motions of the CMC joint are not related to $\theta_{S/P}$ measurements, because the different axes from the rotation of $\theta_{thumb}$ and $\theta_{dorsum}$ are rotated instead; the formula for calculating the S/P angle in actual motion is the same as Equation 11.

## 4. Wearing Method

### 4.1. A Sliding Structure

**Figure 9:**
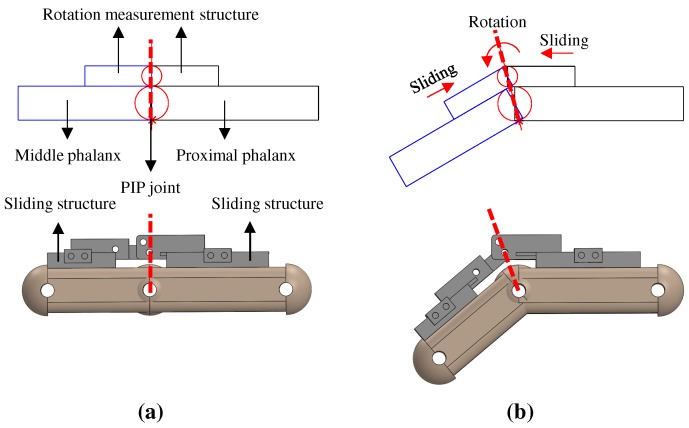
Necessity of sliding structures; (**a**) Initial position; (**b**) Rotated.

The two links of the rotation measurement structure must be parallel to the phalanges during hand motion. The easiest way to accomplish this is to attach the structures to the finger phalanges; however, as it turns out, the two links of the rotation measurement structure should slide over the finger phalanges instead. The necessity of a sliding structure is demonstrated in Figure 9. The upper side of Figure 9 shows a simplified representation of the phalanges and the rotation measurement structure and the bottom side shows the corresponding structure design. In this setup, the finger joint is assumed to be a circular disk, and the phalanges rotate about the disk’s center. The rotation measurement structure is constrained to be parallel to each finger phalanx. In Figure 9a, the rotation axis of the rotation measurement structure is located directly above the center of the finger joint; a dotted line connects the center of the finger joint and the center of the rotation measurement structure. As the finger joint flexes, as shown in Figure 9b, the axis of the rotation measurement structure should move forward to remain parallel to each phalanx. This requires the rotation measurement structure to slide over the finger phalanges. Therefore, a hook-type sliding structure is incorporated into our design. The rotation measurement structure has grooves corresponding to the hook shape of the sliding structure to facilitate sliding motion.

### 4.2. An Underjoint Structure and a Rubber Band

**Figure 10:**
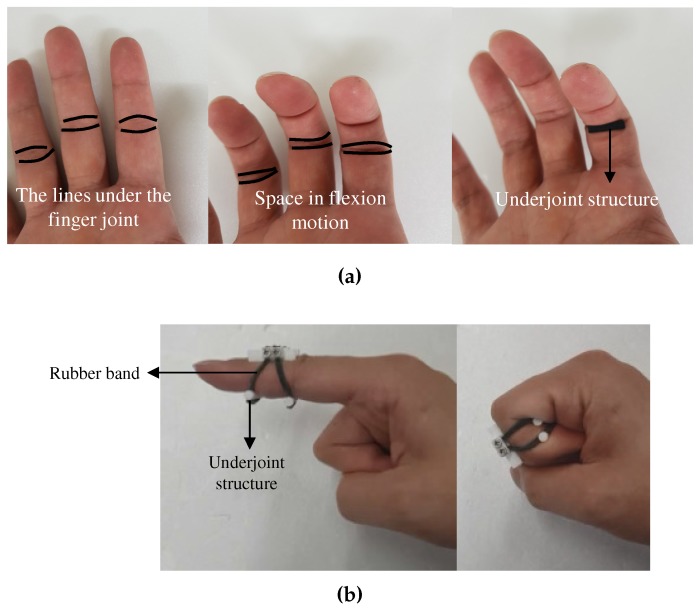
The proposed wearing method using the underjoint structure and rubber band; (**a**) Observation of the space under the finger joint; (**b**) The effectiveness of the underjoint structure in full flexion motion.

With respect to wearability, the proposed system should satisfy two requirements; (1) the sliding structure should be fixed on the finger phalanges during the hand motion, and (2) users with various finger sizes should be able to wear the proposed system. We have considered the characteristics of the lower part of the finger joint during finger motion. Figure 10a shows the lower part of the finger when the finger is flexed. As the finger is flexed, the lower part of the finger joint becomes folded, and the flesh under the phalanges is concentrated in the middle of the phalanges. In particular, we consider that the lower part of the finger joint could be a stationary point for fixing the sliding structure, since the lower part has an empty space during hand motion. After trial and error, as shown in Figure 10b, the maximum flexion of the finger could be achieved by fixing a 3 mm diameter bar at the lower part of the finger joint.

The bar under the finger joint is referred to as the “underjoint structure”. This underjoint structure is connected to the sliding structure with rubber bands, allowing the device to fit various finger sizes; the rubber band can fit different thickness of finger and the sliding structure can fit different finger lengths. Additionally, the sliding structure remains in place during hand motion, as shown in Figure 10b.

## 5. Performance Verification

### 5.1. The Index Finger

**Figure 11:**
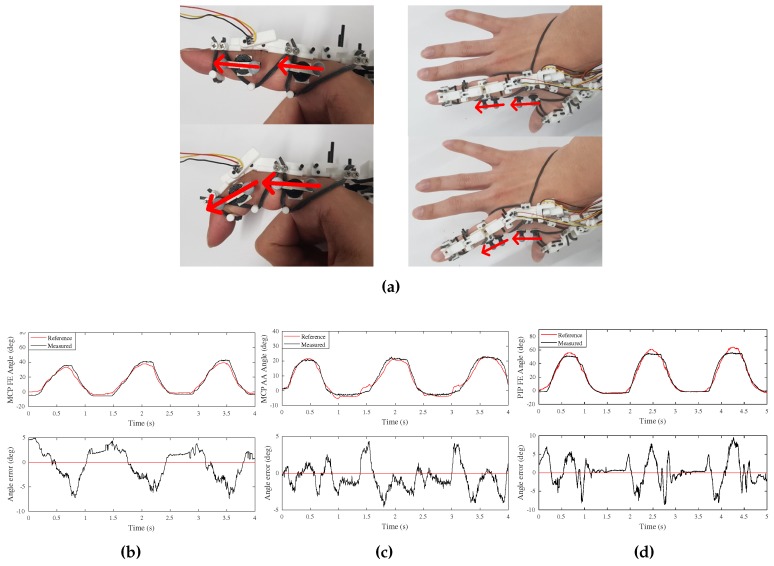
Experimental verification of index finger motion; (**a**) Experimental setup; (**b**) F/E motion of MCP joint; (**c**) Ab/Ad motion of MCP joint; (**d**) F/E motion of PIP joint.

The performance of the proposed hand motion measurement system was verified using an infrared motion capture system. Figure 11a shows the experimental setup of motion measurement performance of the index finger, using the Prime 13 model by Optitrack [4]. Two markers were attached to one finger phalanx around the finger joint to be measured to make a vector, and the actual finger joint angles were compared using the angles between the two vectors rotating around the finger joint.

Total three subjects of different finger sizes participated in the experiment of the index finger. Each motion of F/E of the PIP joint, F/E and Ab/Ad of the MCP joint was performed separately: three or four free finger motions in each trial. The measurement results of three subjects are summarized in Table 3 and one representative result of one subject is shown in Figure 11b–d. The root mean square errors (RMSEs) of the F/E, and Ab/Ad motion of the MCP joint, and the F/E motion of the MCP joint were $2.94^{\circ },1.72^{\circ },$ and $3.37^{\circ },$ respectively, and were similar to the $2.5^{\circ }$ of human finger position-sensing resolution [26].

The main reason for measurement error of the proposed system is the low manufacturing accuracy. As the prototype system was manufactured using a 3D printing technique, the tolerances of the hook in the sliding structure and the groove in the rotation measurement structure were poor, resulting that a rotation measurement structure had less fluidity of movement between structures. More specifically, the measurement performance error increased when the direction of finger motion changed (e.g., when the finger stopped and started to flex and when the finger was bent or stretched). These changes in motion created the greatest friction force in the sliding motion. Thus, poor accuracy of the slider’s tolerance and the large friction force resulted in the maximum measurement error. If the proposed system is made using precision manufacturing processes, rather than a 3D printing technique, we expect that the measurement accuracy will be smaller than the $2.5^{\circ }$ of the human finger position sensing resolution [26]. Also, the measurement accuracy of the PIP joint is less than those of other joints. This is because the length of the middle phalanx is not long enough to properly fix the sliding structure on the finger.

Similar results are shown for various subjects of different hand sizes in Table 3. The results are reasonable because the proposed system was designed to adaptable to various users; (1) measurement principle is not dependent on different hand sizes because it directly measures absolute rotation angle, and (2) the system can be properly worn to users of different hand sizes; the rubber band fits the thickness of the finger and the sliding structure fits the length of the finger. Also, the RMSE of F/E of the PIP joint was larger than those of the MCP joint in each subject; inaccuracy of the PIP joint measurement shows consistency for multiple subjects.

### 5.2. CMC Joint of the Thumb

**Figure 12:**
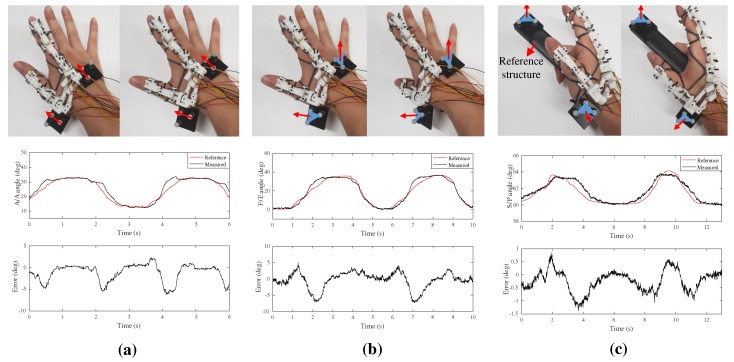
Experimental verification of CMC joint motion; (**a**) Ab/Ad motion of CMC joint; (**b**) F/E motion of CMC joint; (**c**) S/P motion of CMC joint.

In the experiments of the index finger in Figure 11a, two markers were directly attached on the hand: left sides of finger and the dorsum. In the case of the metacarpal of the thumb, it was difficult to attach markers on the hand because the structure covered the upper side of the metacarpal, and much flesh was concentrated on the left side of the metacarpal such that it was highly flexible. Therefore, the thumb experiment was performed by attaching a planar structure consisting of three markers, a square black structure with three markers in Figure 12 next to the sliding structure on the dorsum and the metacarpal.

Experimental settings for the F/E, Ab/Ad, and S/P movements of the CMC joint of the thumb are shown in the upper sides of Figure 12. Before the experiments, we assumed several conditions for the experiments. First, we assumed that the F/E and Ab/Ad axes of the CMC joint were orthogonal [11,23]. The rotation axes used in this section did not match to actual anatomical model. However, note that the objective of the proposed system was to directly use the measured orientation vector. The simple rotation axes, which have been experimentally validated [11], were just selected to verify the performance of the proposed system. Second, we attached markers parallel to the metacarpal, then the motion of the markers was affected by the S/P motion, even during F/E and Ab/Ad motion. Therefore, we did the F/E and Ab/Ad motions after a certain orientation of the CMC joint to minimize the S/P motion as much as possible.

The upper side of Figure 12a shows the experimental setup for Ab/Ad motion, assuming that Ab/Ad motion occurred in the transverse plane. Two markers were selected to make the vector parallel to each phalanx (index finger and thumb), shown as red arrows in Figure 12a. The Ab/Ad angle was calculated as the angle between the two vectors. The upper side of Figure 12b shows the experimental setting for F/E motion. To minimize S/P motion, F/E motion was performed after Ab/Ad motion of the proper angle. The finger was flexed to prevent S/P motion when the F/E motion was at a maximum, as shown in the second left figure of Figure 12b. The normal vectors (red lines) of the planes (blue triangles) formed by the three markers attached to the dorsum and metacarpal were obtained, and the F/E angle was calculated using the angle between the vectors. The upper side of Figure 12c shows the S/P motion setup. In the case of S/P motion, it is difficult to find the reference axis of S/P motion inside the hand, because the metacarpal itself is rotated. A circular bar-shaped structure that could be used as a reference was designed, and S/P motion was performed along the circular structure. The S/P angle was also calculated using the angle between the normal vectors (red lines) of the plane (blue triangles) made by three markers, similar to the experiment for F/E motion. The RMSEs of Ab/Ad, F/E, and S/P motions of the CMC joint were $2.33^{\circ },2.51^{\circ },$ and $0.46^{\circ },$ respectively, which are similar to or less than $2.5^{\circ }$ of the human finger position sensing resolution [26].

In Figure 12a,b, the maximum errors appear near the maximum F/E and Ab/Ad angles. Possible reasons include static friction of the system, or not enough fluidity in the slider due to manufacturing issues. Because the maximum error only occurred at the end of the hand motion, not the beginning, the static friction may not be the main reason. As the maximum errors occurred at similar angles during the hand motion, we conclude that the fluidity in the slider between the dorsum and the thumb due to poor manufacturing quality may be a plausible reason for the measurement error.

In addition, S/P motion had a short range of $4^{\circ }$ because the range, in which S/P motion can be performed along the surface of the cylinder, is short. Lastly, measurement errors in the experiments for the CMC joint seemed relatively low. The measurement errors more than $2^{\circ }$ were measured in the index finger experiment, despite the use of only one rotation measurement structure. However, the errors for the CMC joint were similar to or less than those errors, even though a total of six sensors (i.e., three sensors for calculating the position of one point) were used to measure the motion of the CMC joint. The three sensors used to measure the position of one point include a commercial rotary Hall sensor, the proposed rotation measurement structure, and a linear distance measurement structure (i.e., slider) using the linear Hall sensor. The measurement performance of the commercial products (the rotary Hall sensor) was better than that of the proposed rotation measurement structure. The measurement accuracy of linear distance measurement was also better than that of the proposed structure because the experimentally-obtained relationship between the sensor value and distance was directly used. Also, the change in the angle of the proposed rotation measurement structure during thumb CMC joint movement was very small. To sum up, the rotary Hall sensor and the distance measurement of the slider had better measurement accuracy, and the measurement error of the rotation measurement structure had little effect on position calculations, resulting in a better performance for thumb CMC joint motion than for the index finger.

**Figure 13:**
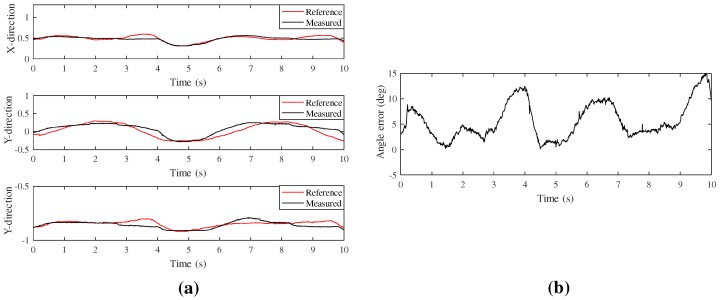
Experiment of the circumduction motion; (**a**) Orientation in the direction of *x-, y*- and *z*-axes; (**b**) Overall error of the orientation.

We conducted another experiment on circumduction motion, which is the combination of all joint angles of the CMC joint. Experimental setup was similar to that of Figure 12a. The vector of the red arrow on the thumb was measured directly by the motion capture system and was compared with the calculated vector of the proposed system. Figure 13 shows the comparison of the orientation vector itself during the circumduction motion. As this motion is the combination of the F/E, Ab/Ad, and S/P motions, the RMSE is bigger than each RMSE of those motions in Figure 12. However, the performance is better than that of previous studies [10,16].

## 6. Conclusions and Future Works

In this paper, we propose a 3D hand motion measurement system that addresses three challenges of existing systems: elimination of the calibration process, universal wearing of a single system, and proper system design to measure the 3D CMC joint motion of the thumb. An absolute rotation measurement structure was developed in a compact configuration using linear Hall sensors to exclude the calibration process. From observations of the lower part of the finger joint as the fixing point, the new wearing method using an underjoint structure and a rubber band provided an adaptable fit to the hand bones for users with various hand sizes.

With respect to measurement of thumb motion, it was very difficult to design the system to decouple F/E and Ab/Ad motions of the CMC joint due to its complex anatomical features. Additionally, the wearing method that fixed the structure on CMC joint while not interfering with the wrist motion was also difficult. However, the proposed system solved these challenges in a different way. As it was difficult to decouple F/E and Ab/Ad motions, instead, we calculated the orientation of the metacarpal itself, which was a combination of F/E and Ab/Ad motions. The obtained result (i.e., the orientation of the phalanx) could be used to realize thumb motion directly in various applications, or F/E and Ab/Ad angles could be calculated from the predetermined kinematic model of the thumb. Lastly, there was no structure on the CMC joint itself; instead, the structure was attached to the metacarpal near the CMC joint using the proposed wearing method, allowing the orientation of the CMC joint to be calculated by the positions of the metacarpal. Thus, the CMC joint was not restricted even in the wrist motion.

Although the proposed system overcame some challenges of existing systems, it still has some limitations. First, precision manufacturing is required to improve the measurement performance. The system itself is designed to guarantee the performance when it is manufactured precisely; the tolerance of the slider between the rotation measurement structure and the sliding structure, and the length change measurement structure including the linear Hall sensor and the magnet, should be accurate. Second, the device has limited wearability, despite being able to fit users of various hand sizes. The tension of the rubber band might make the user feel uncomfortable. Users with very small hands cannot use the system, as the rotation measurement structure has a length of 17–20 mm. Third, the rotation measurement structure at MCP joint of the thumb might not be in a proper alignment because the MCP joint has additional DOF of Ab/Ad motion. Lastly, we did not measure the DIP joint angle because the rotation measurement structures of the PIP and DIP joints were collided on the middle phalanx.

The direction of future works includes easy wearability and additional experiments with various users. It is quite cumbersome to match the underjoint structure correctly. A cradle-based or fabric-based structure may allow for easier wear. In future experiments, we plan to recruit users with various hand sizes to verify the measurement performance by the improved system in terms of wearability and precise manufacturing. Also, repeatability of the proposed system will be conducted.

## Figures and Tables

**Table 1:** DH parameters of $P_{1}$.

i	$\alpha_{i-1}$	$a_{i-1}$	$d_{i}$	$\phi_{i}$
1	0	0	0	$\theta_{1}$
2	$90^{\circ }$	$L_{1}$	0	$90^{\circ }+\theta_{2}$
3	$90^{\circ }$	0	$d_{3}$	0

**Table 2:** DH parameters of $P_{2}$.

i	$\alpha_{i-1}$	$a_{i-1}$	$d_{i}$	$\phi_{i}$
1	0	0	$L_{2}$	$\theta_{1}$
2	$90^{\circ }$	$l_{3}$	0	$90^{\circ }+\theta_{4}$
3	$90^{\circ }$	0	$d_{5}$	0

**Table 3:** Index finger experiments by various subjects. The width and height were measured as the length of the proximal phalanx in the transverse and sagittal planes, respectively. The length of the finger was measured from the MCP joint to the fingertip of the index finger.

		Sub1	Sub2	Sub3
Width, Height, Length (mm)		17, 18, 85	17, 18, 90	23, 23, 100
RMSE (${}^{\circ }$)	F/E of PIP	2.45	3.37	2.68
	F/E of MCP	1.84	2.94	2.34
	Ab/Ad of MCP	2.78	1.72	1.11
